# A216 THE COST-EFFECTIVENESS OF FERRIC DERISOMALTOSE VERSUS FERRIC CARBOXYMALTOSE IN THE TREATMENT OF IRON DEFICIENCY ANAEMIA IN CANADA

**DOI:** 10.1093/jcag/gwae059.216

**Published:** 2025-02-10

**Authors:** R F Pollock, C N Bernstein, S Shaffer

**Affiliations:** Covalence Research Ltd, Harpenden, United Kingdom; University of Manitoba Max Rady College of Medicine, Winnipeg, MB, Canada; University of Manitoba Max Rady College of Medicine, Winnipeg, MB, Canada

## Abstract

**Background:**

In Canada, approximately 0.8% of people have inflammatory bowel disease (IBD), which is estimated to rise by 2.4% annually over the next decade. Iron deficiency anaemia (IDA) is common in people with IBD, who can experience high levels of fatigue that may be alleviated by iron treatment. Intravenous (IV) iron is recommended for people who are intolerant or unresponsive to oral iron or those who require iron rapidly. Ferric carboxymaltose (FCM) and ferric derisomaltose (FDI) are high-dose, rapid-infusion iron formulations approved for treatment of IDA in people with IBD.

**Aims:**

To evaluate the cost-utility of FDI versus FCM when treating IDA in patients with IBD in Canada.

**Methods:**

A published model was adapted to evaluate the cost-utility of FDI versus FCM in Canada. Joint distributions of bodyweight and haemoglobin were used alongside the product monographs (PMs) to model iron need. Iron supply models for FCM and FDI were developed based on posological information in the PMs. Disease-related quality of life (QoL) was modelled using SF-36 data from the PHOSPHARE-IBD randomized trial and a diminishing marginal utility model was used to estimate the effect of iron infusions on QoL. Costs were obtained from Canadian sources aligned with a previous CDA-AMC appraisal of FDI. Price parity between FCM and FDI was assumed, and indirect costs arising from attending IV iron infusions were captured. Analyses were run over 10 years, costs were expressed in 2023 Canadian dollars ($), and future costs and effects were discounted at 1.5% *per annum*. Scenario analyses were performed incorporating fracture incidence based on recent data showing elevated fracture risk after administration of FCM versus FDI.

**Results:**

Relative to FCM, FDI was associated with an additional 0.147 QALYs over 10 years, driven primarily by differences in disease-related QoL (Table). Costs were lower with FDI than FCM ($7,630 versus $9,236), corresponding to a saving of $1,606 per patient over 10 years driven by a reduced number of infusions required to correct iron need with FDI (11.13 versus 14.21 with FCM), and no requirement to monitor phosphate after FDI (Figure). FDI was therefore the dominant intervention. In the fracture scenario analysis, fracture risk was similar before and after treatment with FDI, but was higher with FCM, accompanied by additional costs of $3,086 versus FDI over 10 years ($4,285 versus $1,199).

**Conclusions:**

In people with IBD and IDA requiring IV iron, FDI dominated FCM, reducing costs while improving QoL.

Contributors to QoL over 10 years

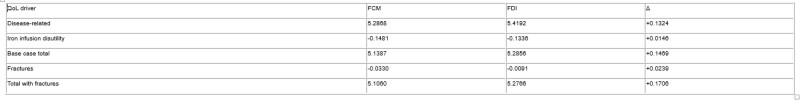

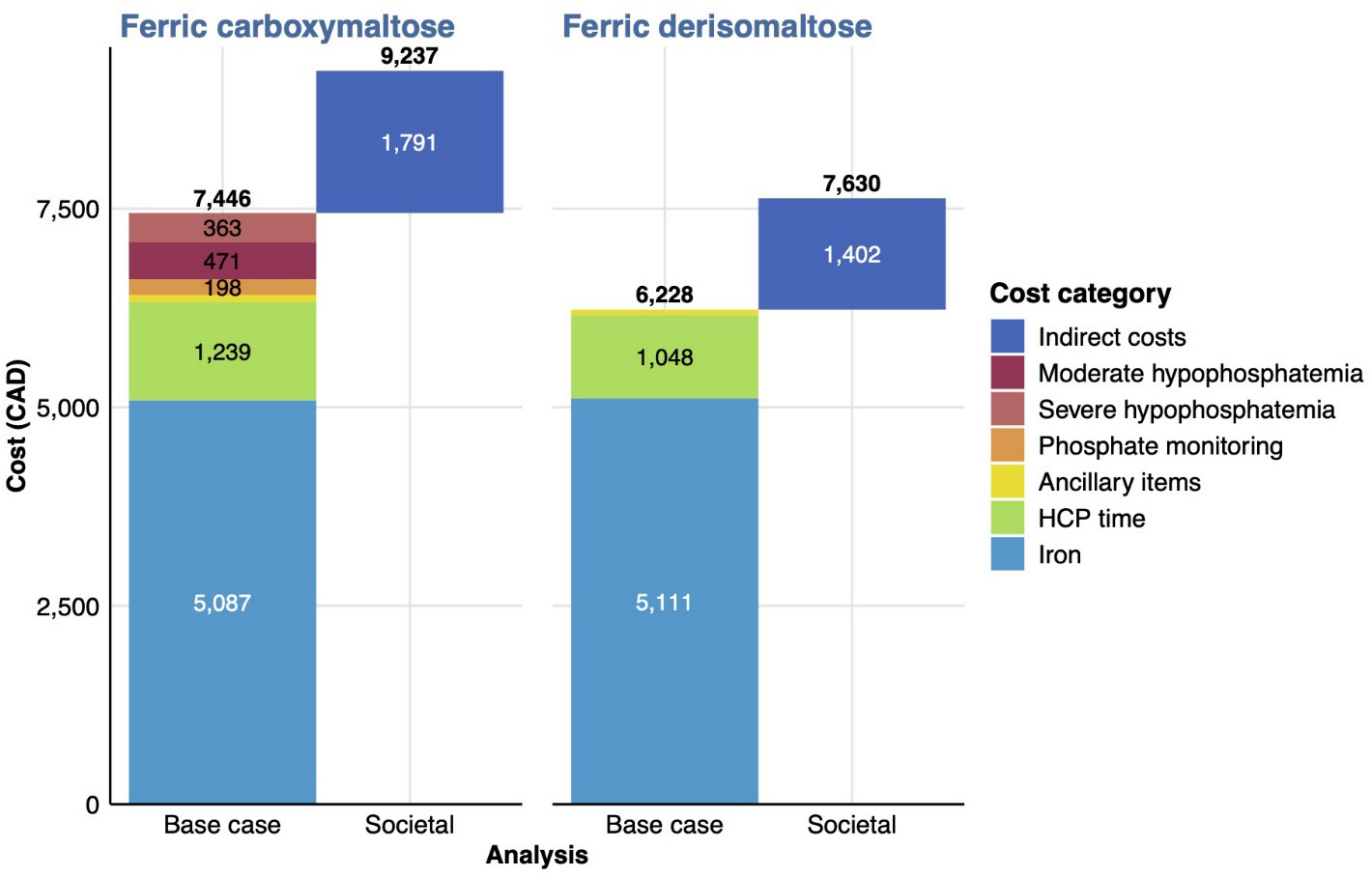

Direct cost drivers in the base case analysis and indirect costs in the societal analysis

**Funding Agencies:**

Pharmacosmos A/S

